# Anti-Nutritional Factors and Protein Dispersibility Index as Principal Quality Indicators for Soybean Meal in Diet of Nile Tilapia (*Oreochromis niloticus* GIFT), a Meta-Analysis

**DOI:** 10.3390/ani12141831

**Published:** 2022-07-19

**Authors:** Shifeng Ma, Hao Wang, Yulong Dou, Xiaofang Liang, Yinhua Zheng, Xiufeng Wu, Min Xue

**Affiliations:** National Aquafeed Safety Assessment Center, Institute of Feed Research, Chinese Academy of Agricultural Sciences, Beijing 100081, China; mashifeng@caas.cn (S.M.); wanghao01@caas.cn (H.W.); blaite_work@163.com (Y.D.); liangxiaofang01@caas.cn (X.L.); zheng-yinhua@163.com (Y.Z.); wuxiufeng@caas.cn (X.W.)

**Keywords:** partial least-squares regression, soybean meal, physicochemical properties, growth, digestibility, *Oreochromis niloticus* GIFT

## Abstract

**Simple Summary:**

Soybean meal is an effective protein source in aquafeeds and is characterized by high protein content, high digestibility, reasonable price, and stable supply. The quality of soybean meal varies with factors such as seed genotype, soil type, planting area, processing conditions, storage conditions, etc. Differences in soybean meal quality may lead to differences in the production performances of animals. For this reason, we investigated the nutrient composition, anti-nutritional factors and physicochemical properties of different soybean meals produced on the same production line with soybeans from different origins, and compared the effects of different soybean meals on the physiological characteristics of Nile tilapia (*Oreochromis niloticus* GIFT). This study identified key indicators that could distinguish the quality of soybean meals, and established a correlation model between quality indicators of soybean meal and the growth performance of Nile tilapia. These results are of great significance for evaluating the quality of soybean meals and promoting the efficient application of soybean meal in aquafeeds.

**Abstract:**

Soybean meal (SBM) is the most important plant protein source in animal feed. This study investigated the characteristics of different SBMs, produced by soybeans from America and Brazil (SBM-A and SBM-B) in 2017–2021 under the same controlled conditions. The effects of different SBMs on the growth performance of Nile tilapia (*Oreochromis niloticus,* GIFT) and apparent digestibility coefficients (ADCs) of nutrients and energy were studied. The results showed that protein dispersibility index (PDI), urease activity (UA), glycinin and fiber were the four primary key indicators for distinguishing the characteristics of the tested SBMs. The meta-analysis results suggested that UA, glycinin, and fiber showed a negative effect on the survival rate (SR) and weight gain rate (WGR) of the Nile tilapia, whereas β-conglycinin, PDI, and nitrogen solubility index (NSI) had a positive effect on the SR and WGR of the fish. The ADCs of dry matter, the gross energy, phosphorus, crude protein, valine (Val), lysine (Lys), histidine (His), serine (Ser), and glutamate (Glu) of the Diet-A group (SBM-A inclusion) were significantly higher than those in the Diet-B group (SBM-B inclusion) (*p* < 0.05). However, no significant difference was found in ADCs of macro-nutrients between the two SBMs (*p* > 0.05). Overall, PDI, UA, glycinin, and fiber were the main indicators reflecting the characteristics of the tested SBMs, and UA, glycinin, β-conglycinin, and PDI had the greatest impact on the growth performance of Nile tilapia in this study. PDI was a more sensitive indicator than NSI for representing the protein quality of SBM.

## 1. Introduction

In most intensive aquaculture systems, feed usually accounts for about 50–70% of total production costs and therefore plays an essential role in the aquaculture industry [1]. Fish meal (FM) is one of the most important protein sources in aquaculture. Due to the limited supply, the future prices of FM will continue to remain high [2]. To maintain the sustainable development of aquaculture, the search for alternative protein sources to FM has become an inevitable requirement. Plant protein from cereals and legumes is a nutritious and economical alternative to FM; it is higher in protein but less palatable, and contains thermostable anti-nutritional factors (ANFs) such as glycinin, β-conglycinin in soybean, and lignin and pectin in all plants [3].

Soybean meal (SBM) is an effective protein source for aquatic feeds and is characterized by high protein content, high digestibility, reasonable price, and stable supply [4]. SBM quality varies with factors including seed genotype, soil type, planting area, processing conditions, and storage conditions [5]. The crude protein content of different batches of SBM varies from 43% to 50% [6], and the amino acid profile also varies, especially the lysine content, which ranges from 5.5% to 6.3% as a percentage of crude protein, and which can be affected by Maillard reaction during heat treatment [7]. A few studies have reported that SBM from different sources significantly affected the nutrient digestibility and growth performance of terrestrial animals, including broilers and pigs [8,9], and also of Pacific white shrimp (*Litopenaeus vannamei*) [10].

Nile tilapia (*Oreochromis niloticus*, GIFT) is one of the most farmed fish in the world, accounting for 8.3 per cent of total finfish production in 2018 [1]. Previous studies have shown that SBM is an effective protein source for Nile tilapia [11,12]. El-Saidy and Gaber (2002) [13] reported that dietary FM could be completely replaced by 55% SBM supplemented with 0.5% L-lysine without adversely affecting the growth performance of Nile tilapia. The apparent digestibility coefficient (ADC) of nutrients in SBM for Nile tilapia were: dry matter, 90.9%; protein, 87.4%; lipid, 92.1%; and fiber, 95.2% [12]. To our knowledge, there has been no study on the effects on the growth of Nile tilapia of SBMs produced by processing soybeans from different origins under the same controlled conditions, nor of the SBMs’ digestibility.

This study aimed to investigate the characteristics of different SBMs produced by processing soybeans from Iowa, USA and Mato Grosso, Brazil in 2017–2021 under the same controlled conditions. The effects were studied of different SBMs on the growth performance of Nile tilapia and the ADCs of nutrients and energy, and a regression model between the indicators of SBMs and the growth performance of Nile tilapia was established by meta-analysis.

## 2. Materials and Methods

During the feeding period, the experimental fish were maintained following the Laboratory Animal Welfare Guidelines of China (Decree No. 2 of Ministry of Science and Technology, issued in 1988).

### 2.1. SBM Samples Collection

Five batches of SBM samples processed with soybeans from Iowa, USA (SBM-A) and Mato Grosso, Brazil (SBM-B) were produced on the same production line and under the same processing conditions during 2017–2021, and were provided by Qingdao Bohai Oil & Fat Co., Ltd., Qingdao, China. The nutrient compositions (crude protein, crude lipid, crude fiber, moisture, ash, and amino acid profile), ANFs (urease activity, trypsin inhibitor activity, glycinin, and β-Conglycinin) and physicochemical properties (protein dispersibility index, nitrogen solubility index) of the SBMs samples were determined.

### 2.2. Experimental Diets

#### 2.2.1. Experiment 1: Growth and ADCs of Tested Diets

During 2018–2020, four growth trials were conducted using the current SBMs. Two isoenergetic and isonitrogenous experimental diets used SBM-A or SBM-B as their main protein sources, referred to as Diet-A and Diet-B, respectively. Growth trials were conducted twice in 2019, and the second test was designed to determine the ADCs of the two diets with 0.1% Yttrium oxide (Y_2_O_3_) as an inert marker. All ingredients were well ground, mixed and formed into 1-mm and 2-mm extruded feed using a Twin-screw extruder (EXT50A, YANGGONG MACHINE, Beijing, China). The extrusion processing conditions were as follows: the pre-conditioning moisture content was 26%, the die temperature was 140 °C and the screw speed was 300 rpm. The extruded feeds were air-dried at room temperature (25 °C) and stored at −20 °C until use.

#### 2.2.2. Experiment 2: ADCs of Tested Ingredients

The preparation and storage methods of the tested diets were the same as in Experiment 1, using SBMs from 2020 as the main protein sources. SBMs were not included in the reference diet. The tested diets consisted of 70% reference diet (RD) and 30% tested ingredients, referred to as TD-A and TD-B. Y_2_O_3_ (0.1%) was used as an inert marker in the tested diets.

The formulations and analyzed nutrient compositions of all the diets are listed in Table 1. The amino acid profiles of the tested diets are reported in Table 2.

### 2.3. Experimental Fish, Feeding and Fecal Collection

The Nile tilapia fingerlings were supplied by Hainan Breeding Station of Beijing Municipal Fisheries Technology Center, Haikou City, China. All fish were acclimated in a recirculation system in the National Feed Safety Assessment Center, Beijing, China and were fed Diet-A for two weeks before the trial started.

In the four repetitions of Experiment 1 during 2018–2020, Nile tilapia (initial body weight: 2.27 g in 2018; 0.99 g in 2019-1; 4.51 g in 2019-2; 57.49 g in 2020) were randomly selected and distributed into conical fiberglass fish tanks (volume: 0.25 m^3^; 256 L, water depth: 80 cm) with 30 fish in each tank and five replicates per treatment. During the experiment, fish were fed to apparent satiation four times per day at 8:00, 11:00, 14:00, and 17:00. The four growth trials had different culture cycles: 60 days in 2018, 70 days in 2019-1, 56 days in 2019-2, and 70 days in 2020. Dead fish were timely removed and their body weight recorded. The feed consumption was recorded daily. Approximately 200 g of fish were randomly collected at the start of the growth experiment and frozen (−80 °C) for the proximate analysis. After the feeding experiment, all fish were weighed and counted to detect the growth performance of the fish after 24 h starvation, including survival rate (SR), weight gain rate (WGR), feeding rate (FR), feed conversion rate (FCR), productive lipid value (PLV) and productive protein value (PPV). Fish body length and weight were recorded separately to compute the condition factor (CF). The viscera and liver weight were recorded individually to compute viscera somatic index (VSI) and hepatosomatic index (HSI). For the determination of tested diets’ digestibility in the 2019-2 trial, feces were collected after 14 days of feeding. During this period, we observed the peak defecation period of the tested fish before collecting feces. The residual feed and feces were removed 30 min postprandial. The feces were collected every 20 min during the peak period of defecation. To ensure the freshness of the feces, the collected feces were transferred to a sealed bag and stored at −20 °C. After collecting a certain amount of feces, the samples were dried in an oven at 70 °C and stored at −20 °C until testing.

Experiment 2: Nile tilapia (initial body weight: 57.49 g) were randomly selected and stocked into fifteen conical fiberglass tanks with 30 fish in each tank and five replicates per treatment. During the experiment, the fecal collection method was the same as in the 2019-2 trial.

The water quality parameters were recorded as follows: water temperature was 24–26 °C, ammonia nitrogen content was lower than 0.5 mg/L, NO_2_- was lower than 0.1 mg/L, dissolved oxygen (DO) was not less than 6.5 mg/L, and pH was 7.8–8.3. Aeration was supplied for 24 h per day. The photoperiod was 14 L/10 D.

### 2.4. Chemical Analysis

Urease activity (UA), trypsin inhibitor activity (TIA), glycinin and β-conglycinin were determined using commercial kits (Beijing Longkefangzhou Bioengineering Institute, China). The nitrogen solubility index (NSI) and protein dispersibility index (PDI) were determined following the method of Araba and Dale (1990) [14] and Iwe et al. (2001) [15]. The detection method for NSI was as follows: the SBM samples were ground and passed through a 0.5-mm screen. The ground SBM sample (1.5 g) was mixed with 75 mL of 0.2% potassium hydroxide solution and shaken for 20 min. The suspended samples were centrifuged at 1200× *g* for 15 min. The protein content of the supernatant was detected by the Kjeldahl method (KjeltecTM 2300 Unit, Foss, Hillerød, Denmark). The NSI was expressed as the percentage of protein dissolved in potassium hydroxide solution compared to the total protein content of the sample. The PDI was determined according to the following method: the SBM samples were milled and passed through a 1-mm sieve. The fine powder (20 g) was dispersed in 300 mL of distilled water. The dispersion was whipped at 10,000 rpm for 10 min using a high-speed disperser (FA25, FLUKO, Shanghai, China). After the suspension was allowed to settle for 10 min, 40 mL of the supernatant was transferred into a 50 mL centrifuge tube and centrifuged at 1300× *g* for 10 min. The protein content of the supernatant was detected using the Kjeldahl method. The PDI was expressed as the percentage of soluble protein compared to the total protein content of the sample. The dry matter, ash, crude protein and crude lipid were analyzed in duplicate following the method of AOAC (2006) [16]. The dry matter was measured at 105 °C. Ash was determined by burning the samples in a muffle furnace (CWF1100, Carbolite, Derbyshire, UK) at 550 °C for 16 h. Crude protein was detected by the Kjeldahl method, and the content was calculated by multiplying the nitrogen content by 6.25. Crude lipid content was determined by acid hydrolysis (Soxtex System HT 1047 Hydrolyzing Unit, Foss, Hillerød, Denmark) and Soxhlet extraction (Soxtex System 1043, Foss, Hillerød, Denmark). The filter bag method was used to determine crude fiber. Gross energy was measured by using an IKAC2000 Calorimeter (C2000, IKA, Germany). The Y_2_O_3_ and total phosphorus in the feeds and feces were tested by inductively coupled plasma atomic emission spectrometry (ICP-OES; JY38S, JobinYvon, Chilly Mazarin, France), and the amino acid contents in the SBMs, diets and feces were determined in the laboratory of Evonik Degussa China Co., Ltd. (Beijing, China) following the standard wet chemistry method. The diets, ingredients and feces were hydrolyzed in 6 N HCl at 110 °C for 24 h. After pretreatment, all samples were analyzed using an Agilent-1100 amino acid analyzer (Agilent Technologies Co., Ltd., Santa Clara, CA, USA).

### 2.5. Imaging Procedure and Photo Processing

The photographs of tested soybeans and SBMs were taken with the same camera (D90, Nikon, Japan) and with the same parameters. The photographs were processed with image analysis software ImageJ (HIH, MD, USA) by analyzing the Red Green Blue (RGB) histograms. RGB pixels were converted to brightness values (Intensity) by using the following formula: Intensity = 0.299 × red + 0.587 × green + 0.114 × blue [17]; lower values meant darker colors.

### 2.6. Calculation and Statistics

The data from the two treatment groups in the same batch of experiments were compared using an independent *t*-test. Significant differences were indicated when *p* < 0.05. The data from different batches for growth performance and morphometric parameters were divided by the maximum value of each batch to calculate the normalized value [18]. Normalized values were then analyzed by one-way ANOVA and Duncan’s multiple range testing to obtain the significant differences between different groups (*p* < 0.05). The data analysis was conducted by IBM SPSS statistics, version 19.0 (IBM Corp., Armonk, NY, USA). The meta-analysis included principal component analysis (PCA), cluster analysis and partial least-squares regression (PLS). The entire dataset was normalized by computing the standard scores prior to analysis, while some variables balanced in the formulations (such as protein, lipids, and amino acids) were excluded from the analysis. To reduce the dimensions and to group different SBM sources, PCA and cluster analysis were conducted on the chemical composition and physicochemical properties of the SBMs by using SIMCA Software package, version 13 (Umetrics, Umeå, Sweden). The importance of extracted PC was derived from SIMCA with built-in cross-validation. Then, PLS analysis was performed using the nutrient compositions, ANFs and physicochemical properties of the tested SBMs (*X*-variables) and growth performance of Nile tilapia (*Y*-variables). The number of significant PLS components was automatically selected based on a statistic (Q^2^). Q^2^ is the cross-validation correlation coefficient, known as the predictive power of the model. The multiple correlation coefficient (R^2^) is another important parameter that provides an estimate of the model fit. Testing was performed with 200 random permutations to avoid over-fitting the model [19]. Jack-knifing was used to estimate standard errors and confidence intervals for the data [20]. The variable importance in the projection (VIP) values for PLS models were used to reflect the contribution of each variable to the model. The VIP scores were calculated as the weighted sum of squares of the PLS weights, w*, taking into account the amount of *Y* variance explained by each extracted latent variable. If the VIP value exceeded 1, it meant the *X* variable was “important” [21].

## 3. Results

### 3.1. Nutrient Compositions and Physicochemical Properties of Tested SBMs

**Figure 1:**
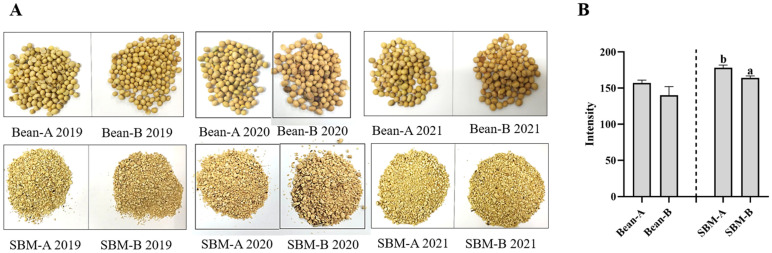
(**A**) The appearance of Bean-A and Bean-B in 2019–2021, and their corresponding soybean meals (SBMs); (**B**) the color intensity of tested soybeans and SBMs. Values marked with “a and b” are significantly different, (*t*-test; *p* < 0.05), means ± SEM (standard error of treatment mean).

The nutrient compositions and physicochemical properties of the tested SBMs are shown in Table 3 and Table 4. There was no significant difference in macro-nutrient and amino acid compositions between SBM-A and SBM-B (*p* > 0.05). The UA of SBM-A was significantly lower than that of SBM-B (*p* < 0.05), while other ANFs and physicochemical properties had no significant difference. The appearance of soybeans from Iowa, USA (Bean-A) and Mato Grosso, Brazil (Bean-B) and their corresponding SBMs produced on the same production line in 2019–2021 are shown in Figure 1A. The color of the SBM-B was darker than that of the SBM-A. The intensities of the photographs are shown in Figure 1B. The results show that the mean of the intensity of SBM-As was significantly higher than that of the SBM-Bs (*p* < 0.05).

**Figure 2:**
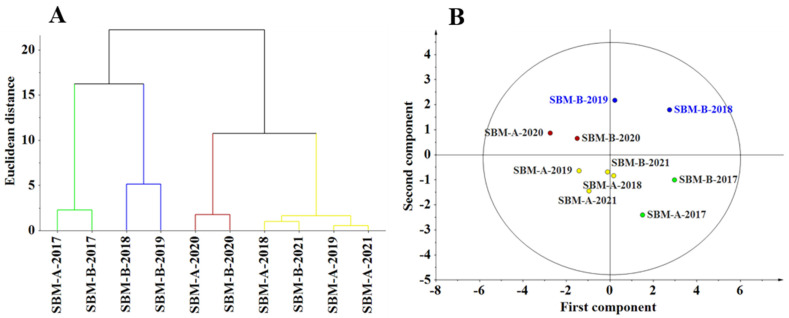
(**A**) Dendrogram of Cluster analysis (grouping of SBM sources base on their chemical composition and physicochemical properties); (**B**) score plot of PCA (grouping of SBM sources based on their chemical composition and physicochemical properties for component 1 (43% of variation) and component 2 (25% of variation)).

The dendrogram generated by the cluster analysis indicates classification of the 10 sources of SBM into four main groups (Figure 2A), which was also shown in the score plot of PCA (Figure 2B). The SBM-A-2017 and SBM-B-2017 were clustered into one group, the SBM-B-2018 and SBM-B-2019 were clustered into another group, the SBM-A-2020, SBM-B-2020 were clustered into a third, whereas the SBM-A-2018, SBM-A-2019, SBM-A-2021 and SBM-B-2021 comprised the fourth group. The PCAs of chemical composition and physicochemical properties of the SBM sources and their loading are reported in Table 5. The first three PCs explained 83.6% of the total sample variance. Based on the loading values, PC1 represented PDI (−0.5042), PC2 represented UA (0.5377) and glycinin (0.5880), whereas PC3 represented fiber (0.8054).

### 3.2. Growth Performance and Morphometric Parameters

The growth performance and morphometric parameters of the four growth experiments carried out in 2018–2020 are shown in Table 6. In the growth trial in 2018, the final body weight (FBW), SR, WGR, and PPV of Nile tilapia in the Diet-A group were significantly higher than those in the Diet-B group (*p* < 0.05), whereas the FCR was significantly lower than that in the Diet-B group (*p* < 0.05). In the 2019-1 growth trial, the FBW, WGR, VSI, and HSI of Nile tilapia in the Diet-A group were significantly higher than those in the Diet-B group (*p* < 0.05). In the 2019-2 growth experiment, the FBW in the Diet-A group was also significantly higher than that in the Diet-B group (*p* < 0.05), whereas the FCR and FR were significantly lower than those in the Diet-B group (*p* < 0.05). In the growth experiment in 2020, there was almost no difference in morphometric parameters and growth performance, except that the PLV and HSI in the Diet-A group were significantly lower than those in the Diet-B group (*p* < 0.05)

The normalized values of growth performance and morphometric parameters are presented in Table 7. There was no significant difference in SR and CF between treatment groups (*p* > 0.05). The WGR in the Diet-A group in 2018 was significantly higher than those in the Diet-B group in 2018, the two Diet-B groups in 2019, and the Diet-A group in 2020 (*p* < 0.05). The Diet-A group in the 2019-2 growth trial had the lowest average FCR, and it was significantly lower than those in the 2018 Diet-B group, the 2019-1 Diet-A group, the 2019-2 Diet-B group, and the Diet-A group in 2020 (*p* < 0.05). The 2019-1 Diet-B group had the lowest FR, significantly lower than those in the Diet-B groups in 2018, 2019-2 and 2020, and the Diet-A group in 2020 (*p* < 0.05). The PPV in the Diet-A group in 2018 was significantly higher than that in the Diet-B group in 2020 (*p* < 0.05), and there was no significant difference between the other treatment groups (*p* > 0.05). The PLV in the Diet-B group in 2020 was significantly higher than in the Diet-B group in 2018 and the two 2019-1 groups (*p* < 0.05). The VSI in the 2019-1 Diet-B group was significantly lower than in the other treatment groups (*p* < 0.05). The HSI of the Diet-B group in 2020 was the highest, significantly higher than those in the two treatment groups in 2018 and the 2019-1 Diet-B group (*p* < 0.05). The average WGR of the Diet-A groups was significantly higher than that of the Diet-B groups (*p* < 0.05), whereas there was no significant difference in other indicators (*p* > 0.05).

### 3.3. PLS Analysis

PLS regression analysis was carried out using ash content, fiber content, PDI, NSI, UA, TIA, glycinin, and β-conglycinin as *X*-variables, and growth performance of Nile tilapia as the *Y*-variable. No significant model was obtained when FCR, FR, PPV, PLV, CF, VSI, and HSI were used as *Y*-variables, respectively. When SR and WGR were used as *Y*-variables, a significant model was obtained that explained 81.1% of the variance in growth performance with a Q^2^ of 61.5% (Table 8).

**Figure 3:**
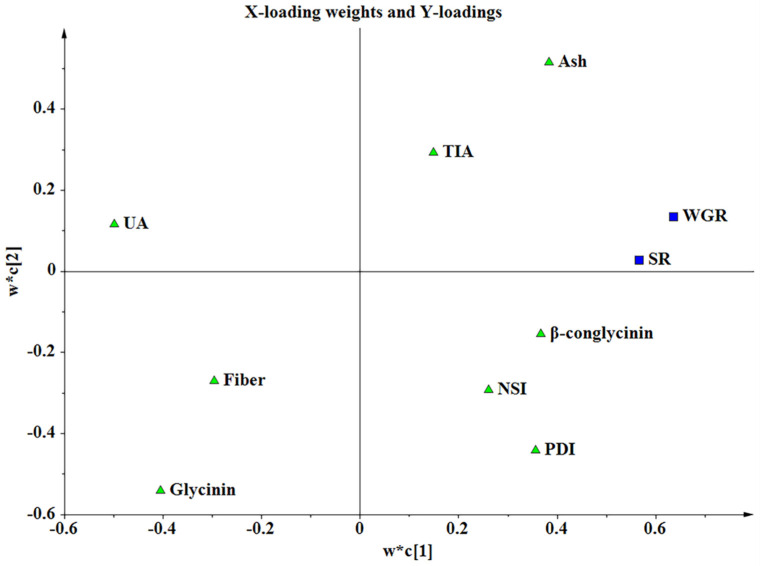
The PLS weights w* and *c* are for the first two components. The w*s are weights that combine the *X* variables; variables with large w* are situated far away from the origin (on the positive or negative side) on the plot; *c* are weights that combine the *Y* variables. This plot shows the correlation structure between *X*, ash, fiber, protein dispersibility index (PDI), nitrogen solubility index (NSI), urease activity (UA), trypsin inhibitor activity (TIA), glycinin, and β-conglycinin, and *Y*, survival rate (SR) and weight gain rate (WGR).

**Figure 4:**
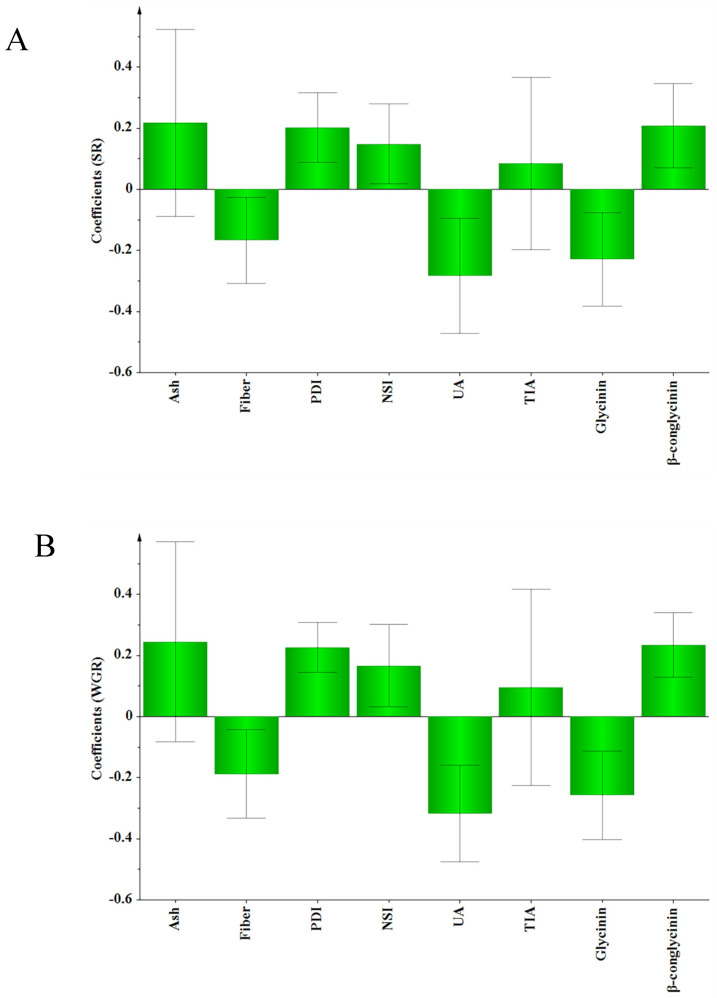
(**A**) The scaled PLS regression coefficients between *X*, ash, fiber, protein dispersibility index (PDI), nitrogen solubility index (NSI), urease activity (UA), trypsin inhibitor activity (TIA), glycinin, β-conglycinin, and *Y*, survival rate (SR); (**B**) the scaled PLS regression coefficients between *X* and *Y*, weight gain rate (WGR). The scaled regression coefficients were weighted. The bars indicate 95% confidence intervals based on jack-knifing.

The weight plot shows that the first dimension was composed mainly of UA, glycinin, β-conglycinin, PDI, and ash (Figure 3). The importance of a given *X*-variable to the *Y*-variable is proportional to its projected distance from the origin in the loading space and corresponds to the PLS regression coefficients [22]. UA, glycinin, and fiber were negatively related to SR and WGR, while PDI, NSI, and β-conglycinin were positively related to SR and WGR (Figure 4). The VIP values of UA, glycinin, β-conglycinin, and PDI were greater than 1 (Table 9). Ash had high VIP (1.085) but was not selected as an important variable because the standard error was large (1.236).

### 3.4. ADCs of Tested Diets and Tested SBMs

The results of apparent digestibility of nutrients and energy in the tested diets and the tested SBMs for Nile tilapia are shown in Table 10. The ADCs of dry matter, crude protein, phosphorus, and gross energy in the Diet-A group were significantly higher than those in the Diet-B group (*p* < 0.05). Among the ADCs of amino acids in the two tested diets, the ADCs of threonine (Thr) were the lowest (88.4%), whereas the ADCs of arginine (Arg) were the highest (97.4% in the Diet-A group and 97.2% in the Diet-B group). The ADCs of valine (Val), lysine (Lys), histidine (His), serine (Ser), and glutamic acid (Glu) in the Diet-A group were significantly higher than those in the Diet-B group (*p* < 0.05). There was no significant difference in ADCs of dry matter, crude protein, phosphorus, and gross energy between the two tested SBMs (*p* > 0.05). Among the ADCs of amino acids in the two tested SBMs, the ADCs of leucine (Leu) were the lowest, 62.6% of the TD-A group and 63.1% in the TD-B group, respectively, whereas the ADCs of methionine (Met) were the highest, 93.2% of the TD-A group and 94.6% of the TD-B group, respectively. The ADCs of phenylalanine (Phe), Glu, and proline (Pro) in the TD-A group were significantly lower than those in the TD-B group (*p* < 0.05).

## 4. Discussion

This is the first time that a direct comparative study has been carried on the nutrient composition, ANFs and physicochemical properties of different SBMs produced on the same production line with soybeans from different origins, to compare the effects of different SBMs on the physiological characteristics of Nile tilapia.

In this study, the PCA results showed that PDI, UA, glycinin, and fiber were the key indicators reflecting the characteristics of SBM-As and SBM-Bs. PDI measures the solubility of proteins in water after high-speed shear treatment, while NSI reflects the solubility of proteins in potassium hydroxide solution. PDI and NSI are important indicators reflecting the degree of thermal denaturation and the molecular status of protein, and are related to the nutritional characteristics of protein materials [23]. The natural protein is denatured when it is subjected to heat treatment under a certain moisture content, and the hydrophobic groups inside the molecular structure are exposed and gather together to form substances of large molecular weight under the action of hydrophobic forces, which results in an obvious decrease in the water solubility of the protein [24]. It has been recommended that well-processed SBM should have a PDI value in the range of 15 to 30% [25]. Batal et al. (2000) [26] suggested that SBMs with a PDI of 45% or less were adequately heat-treated. In our study, all PDI values were <45%, indicating that no SBM was under-processed. The PDI values of SBM-A in 2017 and SBM-B in 2017–2019 were <15%, suggesting that the SBMs may have been overprocessed. We also found that the color of the SBM-B in 2019 was darker than that of the SBM-A. Lee et al. (2021) [27] reported that SBM color could be used to evaluate the heat damage of SBM due to the Maillard reaction. NSI has been widely used as an indicator to estimate the heat damage of SBM [28]. The recommended range of NSI for SBM is 73 to 85% [29]. The NSI values of all SBMs in this study (73.0–84.7%) were within the recommended range, suggesting that the SBMs were well-processed. However, the PCA results showed that the loading value of PDI (−0.5042) was higher than that of NSI (−0.4303), indicating that PDI was more sensitive than NSI for reflecting the characteristics of the tested SBMs. Ibáñez et al. (2020) [30] documented that the PDI and NSI of SBM-A were significantly higher than those of SBM-B. Consistent with the previous study, we also observed that the PDI and NSI of SBM-A (21.4%; 83.1%) were higher than those of SBM-B (15.4%; 79.3%). Normally, soybeans are harvested at higher moisture content in South America, and need to be dried using heated air dryers, whereas many of the soybeans in North America are dried in the field [31]. Thus, soybeans in Mato Grosso, Brazil may have suffered from heat damage before being exported. Indeed, Grieshop and Fahey (2001) [32] showed that soybeans from North America had higher protein solubility than those from Brazil. Since the inactivation of urease in SBM by heating is highly correlated with the inactivation of other ANFs, UA is also a main indicator for indirectly evaluating the quality of SBM in the industry [33]. A high UA indicates the under-processing of the SBM, while a low UA indicates adequate or over-cooking of the meal. Generally, a UA value less than 5 mg N/100 g is considered acceptable for well-processed SBM [34]. García-Rebollar et al. (2016) [35] reported that North American SBM (2.20 mg N/100 g) had a similar UA to Brazilian (2.60 mg N/100 g). In contrast with the previous study, the UA of SBM-A (2.60 mg N/100 g) in our study was significantly lower than that of SBM-B (5.92 mg N/100g), which could be due to the different genetic strains of soybeans. Glycinin is one of the antigenic proteins in soybeans [36]. Per hundred grams of crude protein, the glycinin content (5.69–36.22 g) in this study was within or below the range of values (30–39.2 g) previously reported for SBM [37]. In this experiment, the fiber content of SBM-B (46.6 g/kg) was higher than that of SBM-A (39.6 g/kg), which was consistent with previous studies [35]. The difference in fiber content of the SBMs depended on the geographic area and the season where the soybeans were grown [38].

Differences in the nutritional quality of SBMs may lead to differences in the production performances of animals. Wang et al. (2011) [39] compared the effects of SBM in America, Brazil and India on the growth performance of pigs. The results showed that pigs fed diets containing North American SBM had higher FBW and lower FR than those fed diets containing Brazilian or Indian SBM. In our study, similar results for the Nile tilapia were obtained. Compared with the Diet-B groups, the Diet-A groups had better growth performance, except for the growth trial in 2020. Meta-analysis is a useful method for comparing and synthesizing results from different studies. In our study, meta-analysis was performed to test the relationship between the characteristics of the tested SBM and fish growth performance. The results suggested that UA was the most important indicator determining the growth performance of fish in the present study. UA of the SBMs used in the growth trials varied from 2.24 to 8.28 mg N/100 g, showing a negative effect on the SR and WGR of the Nile tilapia. The meta-analysis also suggested that the glycinin content was negatively correlated with the SR and WGR of the Nile tilapia, which may be because the glycinin caused intestinal oxidative damage and destruction of the intestinal physical barrier. This effect has previously been observed to decrease the growth performance of juvenile Jian carp (*Cyprinus carpio* var Jian) [40]. The meta-analysis suggested that fiber content was negatively correlated with the SR and WGR of Nile tilapia in our study. High crude fiber content reduced feed digestibility in tilapia [41], further retarding the growth performance. β-conglycinin is a major feed allergen; Zhang et al. (2013) [42] reported that dietary inclusion of 80.0 g/kg β-conglycinin reduced the specific growth rate, feed intake, and feed efficiency of juvenile Jian carp, but did not affect the survival of the fish. Contrary to the previous study, the meta-analysis revealed that β-conglycinin had a positive effect on the SR and WGR of Nile tilapia, which could be due to the different fish species. In the present study, the meta-analysis suggested that PDI and NSI had a positive effect on the SR and WGR of Nile tilapia. The low PDI and NSI values could reflect a high incidence of Maillard reaction, ultimately leading to the formation of indigestible Lys-sugar complexes [30]. The results of the 2020 growth experiment showed no obvious differences in the growth performance of the two groups, which may be attributed to the suitability of the processed SBMs (with high PDI and NSI) in their diets. The VIP values reflect the importance of the variables, both for explaining *X* and for correlation to *Y*. The VIP general list shows that UA, glycinin, β-conglycinin, and PDI were the most important *X* variables, each processing a threshold value > 1. These four indicators had the greatest impact on the growth performance of Nile tilapia in our study.

Fish growth is closely related to the digestibility of diet, especially the digestibility of protein [43]. In this study, the ADCs in the Diet-A group for dry matter, gross energy, and phosphorus were significantly higher than those in the Diet-B group. Yang et al. (2009) [44] reported that the energy digestibility of plant products tends to be inversely proportional to the fiber content of the material. The lower ADC of gross energy in the Diet-B group could be due to the higher fiber content in the SBM-B-2019. The high content of fiber could increase the flow rate of chyme in the digestive tract, reducing the contact time between chyme and endogenous digestive enzymes, further reducing the utilization of nutrients [45]. The lower ADC of dry matter in the Diet-B group may be due to the digestion-resistance of glycinin. Consistent with our study, Li et al. (2017) [46] documented that dietary glycinin inclusion (120 g/kg) decreased the ADC of dry matter in juvenile turbot (*Scophthalmus maximus* L.). Phosphorus is an essential component of the fish endoskeleton, as well as cell membranes, nucleic acids, and energy-rich compounds. When excessive phosphorus is excreted through urine and feces, it can cause eutrophication of the water body [47]. The lower ADCs of phosphorus in the Diet-B group could lead to the eutrophication of water bodies in commercial aquaculture, which is a serious problem [48]. The ADCs of crude protein, Lys, Val, His, Ser, and Glu in the Diet-A group were significantly higher than those in the Diet-B group, which could be due to the heat damage experienced by SBM-B-2019. However, the NSI values indicated that all SBM samples in our study were within the recommended range (73–85%), while the PDI values showed that the SBM-B-2019 (<15%) could have been overprocessed. These results showed that PDI was more sensitive than NSI for representing the protein quality of SBM. The over-cooked SBM would be further aggravated during the process of feed extrusion, affecting the bioavailability of amino acids [49]. The unavailability of essential amino acids may worse protein quality. The decreased ADCs of Lys, Val, and His in the Diet-B group could be due to that these amino acids having been combined into one or more compounds during the heat treatment process, and their resistance to attack by digestive enzymes [50]. No significant difference was found in ADCs of dry matter, crude protein, phosphorus, and gross energy between the two tested SBMs, which could be due to the suitability of processed SBMs in 2020.

In summary, PDI, UA, glycinin, and fiber are the key indicators reflecting the characteristics of the SBMs produced on the same production line with soybeans from different origins. UA, glycinin, and fiber showed a negative effect on the SR and WGR of the Nile tilapia, whereas β-conglycinin, PDI, and NSI had a positive effect on the SR and WGR of the fish. UA, glycinin, β-conglycinin, and PDI were the most important variables, having the greatest influence on the growth performance of the Nile tilapia in our study. The ADCs of dry matter, gross energy, phosphorus, crude protein, Lys, Val, His, Ser, and Glu in the Diet-A group were significantly higher than those of the Diet-B group, which could be due to the heat damage to SBM-B-2019. PDI was more sensitive than NSI for representing the protein quality of SBM. No significant difference was observed in the ADCs of dry matter, crude protein, phosphorus, and gross energy between the two tested SBMs, indicating that the SBMs in 2020 were well-processed.

## Figures and Tables

**Table 1:** Dietary formulation and nutrient compositions of experiment 1 and experiment 2 (g/kg, as is basis).

Ingredient (%)	Diet-A ^1^	Diet-B ^1^	RD ^2^	TD-A ^2^	TD-B ^2^
SBM-A ^3^		461		300	
SBM-B	461				300
Soy protein concentrate			250	175	175
Corn gluten meal	82.5	82.5	100	70	70
Spray-dried blood cell meal	40	40			
Fish meal	60	60	235	164.5	164.5
Wheat flour	230	230	210	147	147
Wheat middling	35	35	135	94.5	94.5
Fish oil	20	20	30	21	21
Soybean oil	24	24			
Soy lecithin	15	15	15	10.5	10.5
Vitamin and mineral premix ^4^	11	11	11	7.7	7.7
Y_2_O_3_ ^5^			1	0.7	0.7
CaCO_3_	3.5	3.5			
Ca(H_2_PO_4_)_2_	18	18	13	9.1	9.1
Total	1000	1000	1000	1000	1000
Analyzed nutrient compositions (g/kg, as is basis)					
Moisture	65.7	72.2	104.7	107.7	105.2
Crude protein	381.3	379.4	418.9	428.2	434.5
Crude lipid	52.6	52.4	42.9	38.1	35.8
Crude ash	63.3	62.6	73.5	67.5	63.1
Crude fiber	17.3	23.4	12.3	21.3	27.1
Total phosphorus	8.9	8.9	9.7	9	8.7
Gross energy (MJ/kg)	20.16	20.05	18.84	18.79	18.79

^1^ Diets in the growth trial. ^2^ Diets in Experiment 2; RD: reference diet, TD-A: tested diet for SBM-A, TD-B: tested diet for SBM-B. ^3^ SBM-A and SBM-B were both supplied by Qingdao Bohai Oil & Fat Co., Ltd. (Qingdao, China); Soybean protein concentrate was provided by Yihai Kerry Investment Co., Ltd. (Qinhuangdao, China); Corn gluten meal, soy lecithin and soybean oil were supplied by Bohai Oil Co., Ltd. (Qingdao, China); Spray-dried blood cell meal was supplied by Beijing Hongshun Source Biotech Co., Ltd. (Beijing, China); Fish meal was supplied by Pesquera Exalmar S.A.A., Ltd. (Peru); Wheat flour and wheat middling were produced by Nankou Flour Mill (Beijing, China); Fish oil was provided by Jinhai Grain and Oil Industry Co., Ltd. (Qinhuangdao, China). ^4^ Vitamin and mineral premix was supplied by Chengdu Phoenix Feed Co., Ltd. authorized by USSEC. ^5^ Y_2_O_3_ as an inert marker for digestibility determination was only used in the second growth trial in 2019, the formula was balanced by wheat middling.

**Table 2:** Amino acid profiles of tested diets in growth and digestibility trails (g/kg, as is basis).

Amino Acid	Diet-A ^1^	Diet-B ^1^	RD ^2^	TD-A ^2^	TD-B ^2^
Essential amino acid					
Lysine	21.8	21.8	23.8	25.0	25.4
Threonine	13.8	13.8	16.0	16.5	16.9
Valine	19.7	19.9	20.5	20.8	21.2
Isoleucine	14.0	14.1	18.0	18.6	19.1
Leucine	37.9	38.5	36.8	36.0	36.5
Phenylalanine	19.0	19.5	20.1	20.6	21.2
Methionine	6.80	6.80	9.10	8.10	8.10
Tryptophan	13.6	13.6	/	/	/
Arginine	23.8	23.9	24.7	26.9	27.6
Histidine	10.6	10.7	10.4	10.9	10.9
ƩEAA ^3^	181	183	179	183	187
Conditionally Essential Amino Acid					
Proline	22.0	22.4	24.8	23.8	24.7
Glycine	16.3	16.7	19.6	19.6	20.0
Cystine	3.20	3.30	5.20	5.50	5.70
ƩCEAA ^4^	41.5	42.4	49.6	48.9	50.4
Non-essential amino acid					
Alanine	21.6	22.0	24.0	22.7	22.9
Serine	18.2	17.8	19.2	20.1	21.0
Aspartic acid	38.3	38.5	38.6	42.0	43.4
Glutamic acid	74.1	74.7	72.6	75.0	76.9
ƩNEAA ^5^	152	153	154	160	164
ƩAA ^6^	375	378	383	392	401

^1^ Diets in the growth trial. ^2^ Diets in Experiment 2; RD: reference diet, TD-A: tested diet for SBM-A, TD-B: tested diet for SBM-B. ^3^ ∑EAA: sum of essential amino acids. ^4^ ∑CEAA: sum of conditionally essential amino acid. ^5^ ∑NEAA: sum of non-essential amino acids. ^6^ ∑AA: sum of total amino acids.

**Table 3:** Macro-nutrient and amino acid compositions of tested soybean meals (SBMs) processed on the same production line with beans from different origins (g/kg).

Parameters	SBM-A					SBM-B					SBM-A	SBM-B
	2017	2018	2019	2020	2021	2017	2018	2019	2020	2021	Mean ± SEM ^1^	Mean ± SEM
Macro-nutrient compositions												
Moisture	111	101	118	111	104	106	108	113	111	111	109 ± 3.02	110 ± 1.20
Crude protein	455	461	429	439	460	458	460	437	448	457	449 ± 6.28	452 ± 4.28
Crude lipid	15.8	10.3	11.0	10.4	4.60	13.3	10.7	12.4	9.41	8.10	10.4 ± 1.78	10.8 ± 0.95
Ash	63.9	61.5	58.3	58.1	62.2	64.6	61.4	57.9	61.0	61.0	60.8 ± 1.13	61.2 ± 1.06
Crude fiber	43.2	29.9	39.3	47.1	38.5	47.1	28.5	52.1	56.6	48.9	39.6 ± 2.87	46.6 ± 4.81
Essential amino acid												
Lysine	29.2	28.9	27.6	26.5	29.7	28.5	28.6	28.1	26.3	29.2	28.4 ± 0.58	28.1 ± 0.49
Threonine	18.6	18.3	17.4	16.0	18.9	18.3	18.4	17.9	16.7	19.2	17.8 ± 0.52	18.1 ± 0.41
Valine	22.7	22.8	21.2	19.3	23.6	22.8	23.1	21.9	19.8	22.0	21.9 ± 0.76	21.9 ± 0.58
Isoleucine	22.1	20.4	20.1	17.5	22.1	22.4	20.7	21.0	17.7	20.9	20.4 ± 0.85	20.5 ± 0.77
Leucine	35.8	34.7	33.7	34.7	35.9	35.8	35.2	34.9	34.8	35.8	35.0 ± 0.40	35.3 ± 0.21
Phenylalanine	23.8	23.4	22.6	20.7	23.7	24.2	24.1	23.4	20.9	23.7	22.8 ± 0.58	23.3 ± 0.61
Methionine	6.34	6.14	6.09	5.40	6.56	6.11	6.18	6.12	5.30	6.44	6.11 ± 0.20	6.03 ± 0.19
Tryptophan	6.40	6.50	6.06	6.33	6.38	6.40	6.49	6.18	6.39	6.47	6.33 ± 0.07	6.39 ± 0.06
Arginine	35.2	34.6	32.6	31.6	35.0	34.6	34.2	33.4	31.6	34.2	33.8 ± 0.72	33.6 ± 0.54
Histidine	12.6	12.5	11.6	10.7	12.3	12.5	12.6	11.9	10.6	12.2	11.9 ± 0.36	12.0 ± 0.36
ƩEAA ^2^	213	208	199	182	214	212	210	205	184	210	203 ± 5.86	204 ± 5.19
Conditionally essential amino acid												
Proline	22.8	23.5	22.4	17.8	22.5	21.3	27.1	23.1	18.2	23.2	21.8 ± 1.02	22.6 ± 1.44
Glycine	19.9	19.7	19.0	17.6	19.9	19.9	20.0	19.8	17.8	19.6	19.2 ± 0.44	19.4 ± 0.40
Cystine	7.07	6.84	6.49	3.50	6.70	6.62	7.13	6.52	3.40	6.72	6.12 ± 0.66	6.08 ± 0.68
ƩCEAA ^3^	49.8	50.0	47.9	38.9	49.1	47.8	54.2	49.2	39.4	49.5	47.1 ± 2.09	48.0 ± 2.42
Non-essential amino acid												
Alanine	20.6	20.4	19.2	18.1	20.6	20.8	20.9	19.8	18.4	20.5	19.8 ± 0.50	20.0 ± 0.44
Serine	23.8	24.2	22.4	20.9	23.7	22.5	24.2	23.1	20.7	24.3	23.0 ± 0.60	23.0 ± 0.66
Aspartic acid	54.8	54.0	50.5	48.2	53.8	53.9	54.7	52.4	48.8	54.0	52.3 ± 1.26	52.7 ± 1.05
Glutamic acid	84.6	83.0	79.8	77.6	82.7	83.4	84.4	82.3	77.4	83.2	81.4 ± 1.22	82.1 ± 1.23
ƩNEAA ^4^	183	182	171	165	181	180	184	178	165	182	176 ± 3.56	178 ± 3.31
ƩAA ^5^	446	440	418	392	444	440	448	432	395	442	428 ± 10.2	431 ± 9.45
ƩAA/CP	0.98	0.96	0.98	0.89	0.97	0.96	0.97	0.99	0.88	0.97	0.95 ± 0.02	0.95 ± 0.02

Within the same row, means with different superscripts are significantly different (*t*-test; *p* < 0.05). ^1^ SEM: Standard error of the mean. ^2^ ∑EAA: sum of essential amino acids. ^3^ ∑CEAA: sum of conditionally essential amino acids. ^4^ ∑NEAA: sum of non-essential amino acids. ^5^ ∑AA: sum of total amino acids.

**Table 4:** Anti-nutritional factors and physicochemical properties of tested soybean meals (SBMs) processed on the same production line with beans from different origins.

Parameters	SBM-A					SBM-B					SBM-A	SBM-B
	2017	2018	2019	2020	2021	2017	2018	2019	2020	2021	Mean ± SEM ^1^	Mean ± SEM
Urease activity, mg N/100 g	2.56	2.24	2.31	3.11	2.80	5.31	8.28	6.72	5.32	3.95	2.60 ± 0.16 ^a^	5.92 ± 0.74 ^b^
Trypsin inhibitor activity, U/g	21.4	5.91	3.32	4.43	2.89	21.7	5.62	4.17	4.72	2.31	7.59 ± 3.49	7.70 ± 3.54
Glycinin, mg/g	/ ^2^	59.2	53.9	159	29.9	/	70.9	127	110	26.0	75.5 ± 28.6	83.5 ± 22.5
β-Conglycinin, mg/g	/	52.4	57.9	65.4	67.4	/	44.5	46.4	76.5	45.6	60.8 ± 3.46	53.3 ± 7.76
PDI, %	13.8	19.7	21.7	30.1	21.8	10.1	11.9	13.7	22.6	18.5	21.4 ± 2.61	15.4 ± 2.29
NSI, %	81.6	80.7	84.7	83.9	84.4	77.8	73.0	79.8	81.9	83.8	83.1 ± 0.80	79.3 ± 1.86

Within the same row, means with different superscripts are significantly different (*t*-test; *p* < 0.05). ^1^ SEM: Standard error of the mean. ^2^ The symbol “/” means not detected.

**Table 5:** Principal Component Analysis (PCA) of chemical composition and physicochemical properties of tested soybean meals (SBMs) processed on the same production line with beans from different origins.

Variable	PC1	PC2	PC3
Ash	0.3576	−0.3879	0.1988
Fiber	−0.1743	0.0768	0.8054
PDI	−0.5042	−0.0416	−0.0908
NSI	−0.4303	−0.3443	0.0754
Urease activity	0.2685	0.5377	0.1568
Trypsin inhibitor activity	0.3440	−0.2926	0.4286
Glycinin	−0.1970	0.5880	0.1858
β-Conglycinin	−0.4161	−0.0541	0.2345
Eigenvalue	3.43	2.02	1.23
Variance, %	42.9	25.3	15.4
Cumulative, %	42.9	68.2	83.6

**Table 6:** Effects of dietary soybean meal (SBM) prepared with two different soybean sources on the growth performance and morphometric parameters in Nile tilapia with various initial body weights (means ± SEM, *n* = 5).

Parameters	2018		2019-1		2019-2		2020	
	Diet-A	Diet-B	Diet-A	Diet-B	Diet-A	Diet-B	Diet-A	Diet-B
IBW, g ^1^	2.27 ± 0.00	2.27 ± 0.00	0.99 ± 0.00	0.99 ± 0.00	4.51 ± 0.01	4.52 ± 0.01	57.5 ± 0.03	57.5 ± 0.03
FBW, g	37.4 ± 0.38 ^b^	34.1 ± 0.13 ^a^	40.1 ± 1.22 ^b^	35.1 ± 1.03 ^a^	85.9 ± 2.26 ^b^	77.0 ± 2.59 ^a^	247 ± 11.5	264 ± 4.67
SR, %	94.7 ± 1.33 ^b^	88.0 ± 2.00 ^a^	78. 7 ± 2.71	76.7 ± 3.80	96.7 ± 2.36	89.2 ± 5.34	93.8 ± 2.39	96.3 ± 1.25
WGR, %	1537 ± 18.9 ^b^	1342 ± 21.0 ^a^	3289 ± 122 ^b^	2777 ± 140 ^a^	1752 ± 48.0	1455 ± 117	323 ± 22.7	351 ± 9.75
FCR	1.17 ± 0.01 ^a^	1.24 ± 0.02 ^b^	1.10 ± 0.02	1.07 ± 0.02	0.92 ± 0.00 ^a^	0.98 ± 0.02 ^b^	1.20 ± 0.02	1.16 ± 0.01
FR, % bw/d	2.96 ± 0.03	3.07 ± 0.04	2.96 ± 0.06	2.85 ± 0.04	2.95 ± 0.01 ^a^	3.07 ± 0.03 ^b^	2.12 ± 0.02	2.11 ± 0.01
PPV, %	30.0 ± 0.35 ^b^	27.7 ± 0.18 ^a^	29.7 ± 0.81	28.6 ± 0.42	/ ^2^	/	29.3 ± 1.48	28.8 ± 1.49
PLV, %	119 ± 4.32	105 ± 9.87	136 ± 8.23	133 ± 11.3	/	/	111 ± 4.78 ^a^	126 ± 2.17 ^b^
CF, g/cm^3^	2.80 ± 0.04	2.81 ± 0.02	2.30 ± 0.02	2.28 ± 0.05	/	/	2.34 ± 0.01	2.36 ± 0.03
VSI, %	7.03 ± 0.15	7.00 ± 0.15	11.7 ± 0.43 ^b^	9.95 ± 0.35 ^a^	/	/	9.82 ± 0.32	9.83 ± 0.20
HSI, %	1.27 ± 0.07	1.33 ± 0.04	2.36 ± 0.09 ^b^	2.09 ± 0.08 ^a^	/	/	2.60 ± 0.07 ^a^	2.89 ± 0.02 ^b^

The letters “^a^”, “^b^” indicate a significant difference (*t*-test; *p* < 0. 05) between the two groups. SEM: Standard error of the mean. ^1^ IBW: initial body weight, g; FBW: final body weight, g; SR (survival rate, %) = 100 × final fish number/initial fish number; WGR (weight gain rate, %) = (final total weight (g) − initial total weight (g) + dead fish weight (g))/initial total weight (g); FCR (feed conversion rate) = 100 × feed intake (g)/(final total weight (g) − initial total weight (g)); FR (feeding rate, % bw/d) = 100 × feed intake (g)/((initial total weight (g) + final total weight (g) + dead fish weight (g))/2)/days. PPV (productive protein value, %) = 100 × (final total weight (g) × terminal fish protein content (%) − initial total weight (g) × initial fish protein content (%) + dead fish weight (g) × initial fish protein content (%))/(total food intake (g) × feed protein content (%)); PLV (productive lipid value, %) = 100 × (final total weight (g) × terminal fish lipid content (%) − initial total weight (g) × initial fish lipid content (%) + dead fish weight (g) × initial fish lipid content (%))/(total food intake (g) × feed lipid content (%)); CF (condition factor, g/cm^3^) = 100 × (body weight, g)/(body length, cm)^3^; VSI (viscera somatic index, %) = 100 × (viscera weight, g)/(body weight, g); HSI (hepatosomatic index, %) = 100 × (liver weight, g)/(body weight, g). ^2^ The symbol “/” means not detected.

**Table 7:** Effects of dietary soybean meal (SBM) prepared from different soybeans on the normalized values of growth performance and morphometric parameters in Nile tilapia with various initial body weights (means ± SEM, *n* = 5).

Parameters (Normalized)	2018		2019-1		2019-2		2020		Diet-A ^1^	Diet-B ^1^
	Diet-A	Diet-B	Diet-A	Diet-B	Diet-A	Diet-B	Diet-A	Diet-B		
SR	97.9 ± 1.38	91.0 ± 2.07	90.8 ± 3.12	88.5 ± 4.39	96.7 ± 2.36	89.2 ± 5.34	93.8 ± 2.39	96.3 ± 1.25	95.4 ± 1.16	91.3 ± 1.90
WGR	97.1 ± 1.19 ^c^	84.8 ± 1.32 ^ab^	91.6 ± 3.40 ^bc^	77.3 ± 3.89 ^a^	92.8 ± 2.54 ^bc^	77.1 ± 6.20 ^a^	83.5 ± 5.87 ^ab^	90.7 ± 2.52 ^bc^	91.6 ± 1.96 ^B^	82.3 ± 2.16 ^A^
FCR	90.5 ± 0.95 ^ab^	95.6 ± 1.53 ^cd^	94.6 ± 1.91 ^bcd^	91.9 ± 1.41 ^abc^	89.0 ± 0.36 ^a^	94.7 ± 1.85 ^bcd^	96.9 ± 1.90 ^d^	93.5 ± 0.83 ^abcd^	93.0 ± 0.96	94.1 ± 0.75
FR	92.7 ± 0.90 ^ab^	96.3 ± 1.37 ^bc^	95.3 ± 1.79 ^abc^	91.7 ± 1.37 ^a^	94.1 ± 0.43 ^ab^	97.9 ± 0.88^c^	98.9 ± 0.73 ^c^	98.8 ± 0.30 ^c^	95.0 ± 0.77	95.9 ± 0.85
PPV	95.8 ± 1.12 ^b^	88.7 ± 0.57 ^ab^	92.5 ± 2.52 ^ab^	89.3 ± 1.32 ^ab^	/ ^2^	/	88.1 ± 4.46 ^ab^	86.7 ± 4.49 ^a^	92.5 ± 1.69	88.3 ± 1.28
PLV	92.3 ± 3.36 ^ab^	81.7 ± 3.43 ^a^	81.7 ± 4.95 ^a^	79.7 ± 6.80 ^a^	/	/	85.2 ± 3.67 ^ab^	97.1 ± 1.67 ^b^	86.5 ± 2.54	84.5 ± 3.40
CF	96.6 ± 1.23	97.2 ± 0.78	96.6 ± 0.96	95.7 ± 1.92	/	/	96.9 ± 0.52	97.6 ± 1.13	96.7 ± 0.54	96.8 ± 0.78
VSI	94.2 ± 1.98 ^b^	93.7 ± 2.07 ^b^	87.6 ± 3.23 ^b^	74.8 ± 2.64 ^a^	/	/	92.2 ± 2.97 ^b^	92.3 ± 1.87 ^b^	91.3 ± 1.67	86.6 ± 2.72
HSI	82.6 ± 4.83 ^ab^	86.4 ± 2.73 ^ab^	92.9 ± 3.40 ^bc^	82.0 ± 2.97 ^a^	/	/	89.0 ± 2.44 ^abc^	98.9 ± 0.64 ^c^	88.1 ± 2.39	88.4 ± 2.33

Within the same row, different superscript lowercase letters “^a–d^” denote significant differences among experimental groups based on eight diets (Duncan’s test; *p* < 0.05); different capital letters “^A^” or “^B^” denote significant differences between the mean of Diet-A and Diet-B (*t*-test; *p* < 0. 05). SEM: Standard error of the mean. ^1^ The mean of the growth performance of the four growth trials. ^2^ The symbol “/” means not detected.

**Table 8:** Partial least-squares regression (PLS) models for growth performance of Nile tilapia.

Growth Performance	Significant Components	R^2 1^	Q^2 2^	Permutation Test		
				*Y*-Variables	R^2^-Intercept	Q^2^-Intercept
SR, WGR ^3^	1	0.811	0.615	SR	0.280	−0.139
				WGR	0.253	−0.202

^1^ R^2^, multiple correlation coefficients. ^2^ Q^2^, cross-validation correlation coefficient. ^3^ SR, survival rate; WGR, weight ga rate.

**Table 9:** Variable importance in the projection (VIP) general list.

Var ID (Primary)	VIP
Urease activity	1.410
Glycinin	1.144
Ash	1.085
β-conglycinin	1.040
Protein dispersibility index	1.009
Fiber	0.835
Nitrogen solubility index	0.741
Trypsin inhibitor activity	0.424

**Table 10:** Apparent digestibility of nutrients and energy in the tested diets and the tested soybean meals (SBMs) for Nile tilapia (means ± SEM. *n* = 5).

Parameters	ADCs of Test Diets (2019-2)		ADCs of Test SBMs (2020)	
	Diet-A	Diet-B	TD-A	TD-B
Dry matter ^1^	80.2 ± 0.11 ^b^	79.0 ± 0.13 ^a^	78.8 ± 1.67	79.0 ± 1.86
Crude protein	92.7 ± 0.11 ^b^	92.2 ± 0.14 ^a^	93.1 ± 0.61	94.0 ± 0.54
Phosphorus	68.2 ± 0.43 ^b^	67.0 ± 0.32 ^a^	40.9 ± 4.55	40.4 ± 6.11
Gross energy	84.1 ± 0.10 ^b^	82.9 ± 0.13 ^a^	83.9 ± 0.78	84.5 ± 0.78
Apparent digestibility of essential amino acids				
Threonine	88.4 ± 0.37	88.4 ± 0.22	79.8 ± 1.66	79.8 ± 1.24
Valine	93.1 ± 0.06 ^b^	92.9 ± 0.05 ^a^	80.8 ± 0.42	82.1 ± 0.94
Methionine	94.4 ± 0.13	94.3 ± 0.18	93.2 ± 0.27	94.6 ± 0.60
Isoleucine	93.4 ± 0.10	93.4 ± 0.06	80.8 ± 0.97	80.6 ± 1.25
Leucine	94.0 ± 0.15	94.0 ± 0.04	62.6 ± 2.86	63.1 ± 2.98
Phenylalanine	93.6 ± 0.13	93.5 ± 0.04	80.3 ± 0.80 ^a^	84.0 ± 0.77 ^b^
Lysine	96.3 ± 0.09 ^b^	95.9 ± 0.08 ^a^	90.1 ± 0.45	90.7 ± 0.64
Histidine	95.6 ± 0.03 ^b^	95.3 ± 0.03 ^a^	93.3 ± 0.48	93.9 ± 0.35
Arginine	97.4 ± 0.06	97.2 ± 0.12	90.5 ± 0.79	92.5 ± 0.64
Apparent digestibility of non-essential amino acids				
Aspartic acid	95.6 ± 0.10	95.4 ± 0.07	82.2 ± 1.13	83.2 ± 0.80
Serine	94.5 ± 0.08 ^b^	94.0 ± 0.09 ^a^	87.5 ± 0.44	88.1 ± 0.50
Glutamic acid	97.3 ± 0.04 ^b^	97.1 ± 0.04 ^a^	69.4 ± 1.06 ^a^	74.2 ± 1.54 ^b^
Glycine	91.9 ± 0.16	91.4 ± 0.15	82.1 ± 0.98	83.5 ± 0.50
Alanine	93.4 ± 0.09	93.4 ± 0.05	78.5 ± 0.67	80.8 ± 1.04
Proline	94.4 ± 0.15	94.2 ± 0.12	76.6 ± 1.75 ^a^	84.6 ± 1.54 ^b^
Cysteine	92.8 ± 0.28	92.2 ± 0.30	93.8 ± 0.38	93.9 ± 0.17

The letters “^a^” and “^b^” indicate a significant difference (*t*-test; *p* < 0. 05) between the two groups. ^1^ ADC of dry matter in tested diet (%) = 100 × [1 − marker (Y_2_O_3_) in diet / marker (Y_2_O_3_) in feces]. ADC of nutrients or energy in tested diet (%) = 100 × [1− (nutrients or energy of feces/nutrient or energy of diet) × (marker (Y_2_O_3_) in diet / marker (Y_2_O_3_) in feces)]. ADC of dry matter in tested SBM = (ADC of dry matter in diet with test ingredient − 0.7 × ADC of dry matter in reference diet)/0.3; ADC of nutrients or energy in in diet with test ingredient = ADC of nutrients or energy in tested diet + [(ADC of nutrients or energy in tested diet—ADC of nutrients or energy in the reference diet) × (0.7 × (nutrient content of reference diet/0.3) × nutrient content of test SBM)].

## Data Availability

The data presented in this study are available on request from the corresponding author.

## Abbreviations

ADC: apparent digestibility coefficient; ANF: anti-nutritional factor; CF: condition factor; FCR: feed conversion rate; FM: fish meal; FR: feeding rate; HSI: hepatosomatic index; NSI: nitrogen solubility index; PCA: principal component analysis; PDI: protein dispersibility index; PLS: partial least-squares regression; PLV: productive lipid value; PPV: productive protein value; SBM: soybean meal; SR: survival rate; TIA: trypsin inhibitor activity; UA: urease activity; VAI: visceral adipose index; VIP: variable importance in the projection; WGR: weight gain rate.
